# Using the Consolidated Framework for Implementation Research to design and implement a perinatal education program in a large maternity hospital

**DOI:** 10.1186/s12913-021-07024-9

**Published:** 2021-10-11

**Authors:** Sheridan Guyatt, Megan Ferguson, Michael Beckmann, Shelley A. Wilkinson

**Affiliations:** 1 Physiotherapy Department, Mater Misericordiae Ltd, Level 2 Mater Hospital, Raymond Terrace, South Brisbane, Queensland 4101 Australia; 2 Faculty of Medicine, The University of Queensland, Brisbane, Queensland 4006 Australia; 3 Mater Research, The University of Queensland, Brisbane, Queensland 4101 Australia; 4 School of Public Health, Faculty of Medicine, The University of Queensland, Brisbane, Queensland 4072 Australia; 5 Menzies School of Health Research, Royal Darwin Hospital Campus, Darwin, 0810 Australia; 6 School of Human Movements and Nutrition Science, Faculty of Health and Behavioural Sciences, The University of Queensland, Brisbane, Queensland 4072 Australia

**Keywords:** Theory-informed, Implementation, Perinatal education, Co-design, Knowledge translation

## Abstract

**Background:**

Implementation science aims to embed evidence-based practice as ‘usual care’ using theoretical underpinnings to guide these processes**.** Conceptualising the complementary purpose and application of theoretical approaches through all stages of an implementation project is not well understood and is not routinely reported in implementation research, despite call for this. This paper presents the synthesis and a collective approach to application of a co-design model, a model for understanding need, theories of behaviour change with frameworks and tools to guide implementation and evaluation brought together with the Consolidated Framework for Implementation Research (CFIR).

**Method:**

Using a determinant framework such as the CFIR provides a lens for understanding, influencing, and explaining the complex and multidimensional variables at play within a health service that contribute to planning for and delivering effective patient care. Complementary theories, models, frameworks, and tools support the research process by providing a theoretical and practical structure to understanding the local context and guiding successful local implementation.

**Results:**

This paper provides a rationale for conceptualising the multidimensional approach for implementation using the worked example of a pregnancy, birth, postnatal and early parenting education intervention for expectant and new parents at a large maternity hospital.

**Conclusion:**

This multidimensional theoretical approach provides useful, practical guidance to health service researchers and clinicians to develop project specific rationale for their theoretical approach to implementation projects.

*“There is nothing so practical as a good theory”* Kurt Lewin ( [1])^p169^.

## Background

### Implementing change in perinatal care

The perinatal period is a time when the majority of women are engaged with the health care system regardless of age, socioeconomic status or ethnicity [2]. Expectant and new parents are motivated to change behaviours that contribute to their health and wellness and that of their baby [3]. This provides an *opportunity* for the development and delivery of effective, evidence-based education building health literacy and adopting healthy behaviours and lifestyle. Interventions aimed at increasing knowledge, skills and behaviours have been shown to improve outcomes in pregnancy, birth, postnatal recovery and the early parenting period [4–7] and can have lasting benefits to the future health and wellbeing of the entire family in a research setting [8, 9].

However, an evidence-practice gap exists in the delivery of universal perinatal education. Whilst education provided for expectant and new parents is a valued component of the care provided by health care providers throughout pregnancy and following birth it does not meet the intended populations needs with the content and format of education based largely on tradition and not emerging evidence [10, 11]. Expectant and new parents want education that is consistent, timely, practical and gives opportunity for peer interactions [12–14]. Perinatal education does not meet its potential to impact birth outcomes, breastfeeding, ongoing physical and mental health, couple relationships or parenting self- efficacy [15–17]. Perinatal education is a core component of perinatal care, a recognised complex health care paradigm that is not clearly understood [18] and in its various forms in Australia is unregulated, inconsistent and outcomes are unknown [11, 19, 20]. There are barriers to closing this evidence-practice gap across regulatory, professional, organisational and local settings [19].

### Why use implementation science?

Implementing change within this complex system can be addressed through a thoughtful and reasoned approach to developing research methodology that considers the interrelationship between health care providers, expectant and new parents, local infrastructure, systems, policies, and external influences. This change process will require a reasoned, multidimensional approach to realist research incorporating experience-based co-design [19, 21]. Re-imagining, developing, and delivering an effective perinatal education intervention requires an understanding of the complexity of the interrelationships in the local context, application of behavioural and implementation science theory to develop the intervention and plan implementation, and a methodology for ongoing evaluation, improvement and sustainability [22, 23]. There has been increased interest by researchers, funders and health service providers in strategies that improve the adoption of evidence into sustainable patient care. This has prompted the growth in the last two decades of research in healthcare on knowledge translation or implementation science (translating evidence into practice) and incorporating complexity science (understanding systems through the interconnections between the agents within and around systems) into health service research [24].

Implementation science aims to provide a theoretical underpinning to guide the implementation of evidence-based practices sustainably into the health care system. As an emerging science, implementation science pioneers are developing the field by drawing from public health, psychology, and business and then synthesising and testing these theories in their implementation experiences and reviewing published research reporting translation success and failures [25]. The evolving theories, models, frameworks, and tools being developed and tested highlight the range of factors for successful implementation of health care innovations aiming to change efficacy of health care delivery. To facilitate growth in this field consistent reporting of the scientific rationale behind the application of these implementation strategies has seen the development of published reporting guidelines [26–30].

Translating implementation science into clinical research requires an understanding of both the theoretical underpinnings and the rationale for applying these elements in different health care contexts and across the phases of an implementation process. This provides an opportunity to then test, review, consolidate and refine understanding for use across varied contexts. Using a recognised theoretical approach to guide implementation also facilitates the scientific reporting of the rationale and theory underpinning the implementation strategy used in a study.

### Three key features of implementation science

Implementation science’s key premise that makes it a useful theoretical approach for health service research is that it is practical, yet rigorous and systematic. It addresses past failures in translating what is proven to be effective in research into what is realistic, practical, scalable, and transferable in health care settings. Three features of the implementation science approach we identify that make it applicable within health care, particularly the perinatal care setting are:

1. *Implementation science is theory driven, yet pragmatic.* While the methodology promotes a systematic approach it is not prescriptive, meaning that it can be operationalised to be realistically achieved and practically fit local health care contexts. Implementation science recognises the multidimensional complexity of health care (no more so than in perinatal care) and the continuous nature of change and therefore does not present a simplistic, two dimensional or linear process to follow. Instead, implementation science provides frameworks, models and tools that can be blended to address different facets of the implementation process [31] and a common language that provides insight into the process and extends learnings into other contexts.
2. *Engagement matters and enhances success.* Engaging with the breadth of stakeholders facilitates ongoing intervention success through developing an understanding of the implementation enablers and barriers and how they interact in the local context. Active co-design partnership with the people who engage with the health care setting (patients and their carers) contributes to meeting their needs with effective user focussed care. Co-design with health care providers deepens the understanding of and influence within existing clinical networks, cross discipline interaction and informal relationships [23, 24]. In our perinatal care context, the expectant and new parents and frontline perinatal care providers are central to this engagement within the hospital setting where the implementation is taking place.
3. *Behaviour science contributes to implementation success.* Understanding the determinants of behaviour and how these can be influenced has been applied to public health interventions [32], in individual patient care aiming to support behaviour change [7, 33] and more recently to influencing the behaviour of health care providers [34] toward implementation of evidence–based care [35]. Past implementation failures can at least in part be attributed to the complexity of human behaviour, such as the repeated failure to implement e-health technologies such as telehealth [22, 36]. The transition through the perinatal period is an important touchpoint for public health and individual patient care with behaviour science improving efficacy.

In 2017 the Queensland Health Department published recommendations for perinatal education [11]. These recommendations together with an internal hospital review highlighted a need to re-imagine perinatal education in our local context. This paper aims to outline the synthesis of a theoretical, multidimensional implementation science approach to delivering an effective perinatal education intervention. It presents the rationale underpinning a health service research program to design, implement and evaluate perinatal education within perinatal care detailed in Fig. 5. This research is currently underway and will be reported in future publications including evaluation of local needs, program design and health outcomes in pre and post implementation cohorts.

## Methods

**Fig. 1:**
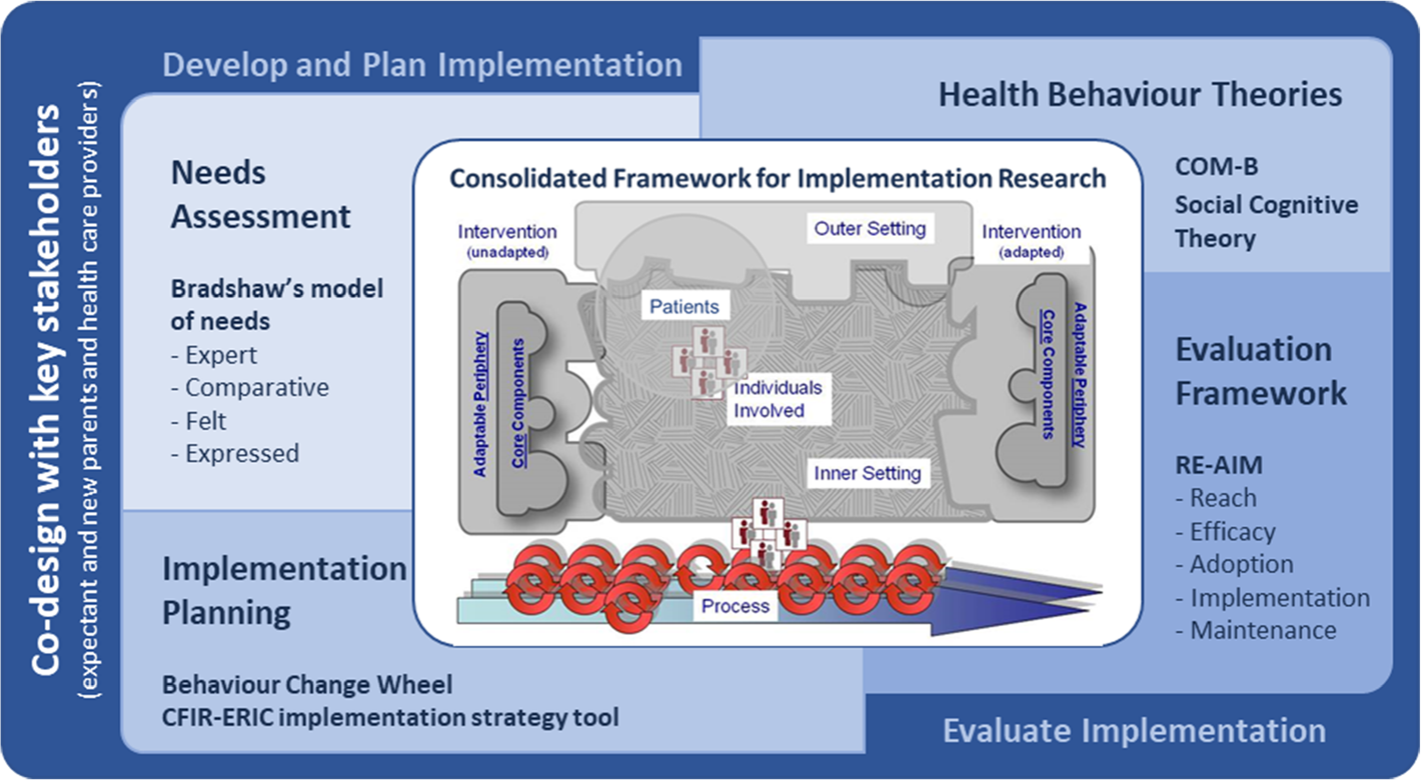
Theoretical approach to the design and implementation of a perinatal education intervention within a large maternity hospital. The central implementation framework is the CFIR [37] which has been modified to include an additional *patients* domain and is surrounded by additional theories, models, frameworks, and tools to illustrate the scientific approach taken for the development, planning, implementation and evaluation of a perinatal education intervention

Implementation science provides a theoretical approach to design and implement perinatal care interventions. We have identified and defined six key elements to implementation science-based research that we have brought together to apply in our health service setting. These are:1. guiding and articulating the implementation process with a central *Determinant framework*; 2. engaging with expectant and new parents through ongoing active *Co-design*; 3. *Understanding needs* in the local setting from different perspectives; 4. an evidence informed *Intervention design* guided by health behaviour theory; 5. to *Plan and execute implementation* using tools to address barriers to success and; 6. *Evaluate and sustain* the intervention in an ongoing cycle of evaluation, review and improvement. This multidimensional implementation science response is summarised in Fig. 1. While presented here in an order this is not a stepwise approach. Element one for example articulates the entire process and co-design (element two) is a part of each stage with evaluation listed as the final element being important to consider from the outset of research design. All elements continue to play a part of ongoing sustainability and future re-iterations of the intervention (see Fig. 5). This seemingly messy, winding and looping multidimensional path is a strength of taking an implementation science approach [31, 38, 39].

Our rationale for bringing these six elements together is described below. This worked example of the design of an implementation project aims to produce a woman and family centred program of perinatal education. It aims to deliver the right education at the right time, to improve health literacy, health behaviour and health and wellbeing outcomes via a multimodal intervention.

There are many published theoretical approaches to each aspect of implementation science and several key questions can guide the selection of which theoretical approach to use; why, who, and what approach [40]. *Why* a specific theoretical approach is selected depends on the purpose of its use and the phase of the implementation process. In this example the ‘why’ is to guide and action elements of the process from conceptualisation, through planning and implementation to an ongoing model of sustainability. *Who* the stakeholders and the research context are will influence the theories, models, frameworks, and tools best suited to different scales within the health system (autonomous individuals, teams, whole organisations or even system wide) and in different contexts (including the degree of stakeholder diversity, complexity of intervention and purpose of the intervention)? Our research is centred around a large tertiary maternity hospital in an urban setting in Queensland, Australia where over 10,000 babies are born each year and the care provided by the multidisciplinary team to pregnant and postnatal women and their support people accessing private and publicly funded maternity care. Understanding the origins and purposes of varied theoretical approaches (theories, models, frameworks and tools) informs *what* approach/s to use as does the supporting resources, ease of understanding and the researchers past experience [31, 39, 40].

Conceptualising and applying these questions in our research started by identifying a suitable determinant framework followed by complementary theories, frameworks, models, and tools that can be operationalised to meet all component needs from intervention development and implementation to evaluation of a perinatal education intervention. The rationale for their selection is detailed below.

### Six elements of implementation science applied within a theoretical approach

#### Determinant framework

A determinant framework defines the determinants of implementation success or failure into types or domains and subtypes or constructs that act as the modifiable variables in the implementation process [25]. A determinant framework provides an overarching lens and a shared understanding of these conceptual constructs from development to evaluation. Several key determinant frameworks have been developed, built on a synthesis of existing research in the field. The determinants follow a similar pattern of domains with some variations in expression between each framework [25, 31, 38, 40]. The iPARIHS (integrated framework for Promoting Action in Research Implementation in Health Services) for example, is designed around the role of facilitator [41], the Non-adoption, Abandonment and barriers to Scale-up, Spread and Sustainability (NASSS) framework has been designed for targeted use with health technology interventions [22], and the CFIR more broadly describes factors that influence implementation by defining theoretical constructs and providing a common language that has been applied across a variety of research contexts [38, 42].

#### Selecting a determinant framework

The CFIR is a comprehensive meta-framework and the central determinant framework used in this theoretical approach (Fig. 1) to plan, implement and evaluate a co-designed perinatal education program in a large maternity hospital. CFIR was chosen by matching the purpose (why) and context (who) of our research with our understanding of the CFIR (what). CFIR is based on a synthesis of existing frameworks and draws together the theory of implementation science with an iterative *active change process* for participatory action research as one of five broad domains - the outer setting, inner setting, the individuals involved, intervention and process [37]. Within each domain there are multiple constructs, which further describe factors within the domain and interactions between domains in a research context. CFIR recognises that interventions can be locally adapted to fit the inner setting with a core non-adaptable component that maintains intervention integrity, and this facilitates transferability to other contexts or settings.

We chose CFIR because: i) CFIR has been tested in a range of health care settings with over 300 peer reviewed articles reporting its application including settings of a similar size and complexity to ours [43], had been effectively applied to other multifaceted, patient focused interventions [44], and does not rely on a specific role of the researcher within the framework [37]; ii) CFIR is a multifaceted framework designed to be applied pragmatically from inception to implementation evaluation [44, 45]; and iii) CFIR has been successfully used with other theories, models, and frameworks [34, 45, 46] including perinatal interventions [47–49]. Additional research support is available from a worldwide community of researchers who have used CFIR and developed, tested and published additional tools and resources [43].

#### Applying the CFIR to perinatal care

We have defined the CFIR domains shown in Fig. 1 in the context of implementing a perinatal education intervention at a large maternity hospital as:

*Intervention:* The perinatal education program (both pre and post implementation). CFIR defines interventions (in our case perinatal education) as made up of *core components* (transferable between settings) and an *adaptable periphery* (adapted to fit the local inner setting). Articulating these components when reporting on intervention design will assist transferability of our research findings.

*Outer setting:* This encompasses external influences in the broader community. The outer setting includes the social and cultural factors within the local community and extends to peak body recommendations, Australian and Queensland Government policy, and key literature findings regarding perinatal education. This includes the general population, but we have separated our patient population of expectant and new parents into a separate domain (described below).

*Inner setting:* A large tertiary maternity hospital in south-east Queensland. The inner setting includes the people, culture, systems, infrastructure, and resources of the hospital.

*Process:* The active change process used. This process cycles through the elements of planning, engaging, executing, and evaluating at micro and macro levels within the inner setting.

*Individuals involved:* The people within the inner setting, in this case maternity health care providers.

In addition to these domains, we included a sixth domain:

*Patients:* Expectant and new parents are separately identified to highlight the central part they play in our context. Using Damschroder’s definition of domains *expectant and new parents* fall within the *outer setting* [37] but in our theoretical approach they are also an active component of the *individuals involved* within the *inner setting* so we have followed the lead of other implementation researchers [44, 50] and separated expected and new parents for independent consideration alongside the original five domains. This will facilitate a woman and family centred approach to perinatal education.

#### Co-design

The second key element and underlying support to research efficacy is the concept of *co-design* - by meaningfully engaging with the end-users of the proposed intervention [51]. The end-users in the case of perinatal education are both expectant and new parents and their health care providers who develop and deliver perinatal education.

**Fig. 2:**
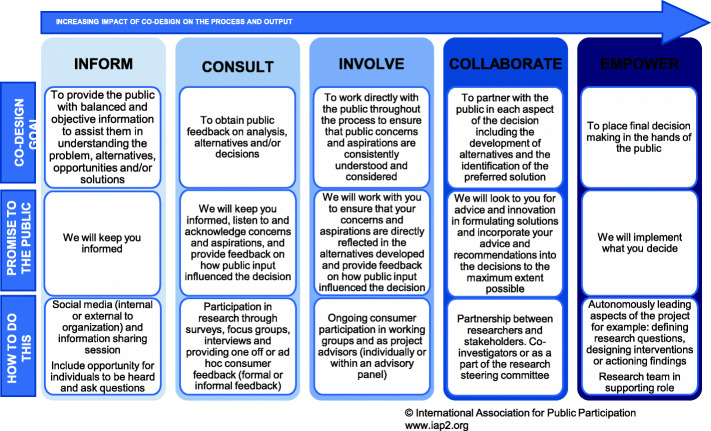
The IAP2 spectrum for public participation [53] including how co-design is actioned in our research

Successful intervention implementation in a real-world perinatal care setting needs to address complexity and requires pragmatism for success. A strong partnership with key stakeholders supports this success through the co-design of i) research processes, ii) the intervention and iii) how this is implemented [18, 21, 52]. Each stage of an implementation project provides opportunity for co-design in partnership with the end-users of the intervention. These end-users are identified as the CFIR domains *individuals involved* and *patients*. In this research context our partnership is with the health care providers who design and deliver perinatal care (including education) and local expectant and new parents. Co-design requires thoughtful consideration and intentional planning. To support this engagement with members of a community the International Association for Public Participation (IAP2) has conceptualised this as a spectrum (Fig. 2) [53].

When applied to research this ranges from having an awareness of the research or intervention, being a participant in the research, actively advising the research team or as co-investigators, up to leading the research team. The IAP2 model can be applied to all key stakeholders including organisational leaders, frontline health care providers and expectant and new parents. Active engagement through co-designing with stakeholders throughout all aspects of the implementation process improves research relevance to the end-users and implementation success. Of note, there is a gap in the literature in recording and evaluating the contribution of co-design in implementation research [51] with reporting guidelines developed to help provide consistency [30].

#### Understanding needs

A pre-implementation stage and essential element of the implementation process is to *understand* the local context. This understanding includes identifying current evidence-practice gaps, benchmarking with others, understanding local stakeholder needs and identifying the enablers and barriers to implementation. A needs assessment is a systematic process that facilitates this understanding of the current state and identifies planning and implementation priorities as well as directing future evaluation [54, 55].

Assessing local need is a recommended part of all community service development and delivery. Community and women’s needs are identified as a priority within global, nationwide and local recommendations and guidelines for perinatal care and perinatal education [11, 56, 57].

**Fig. 3:**
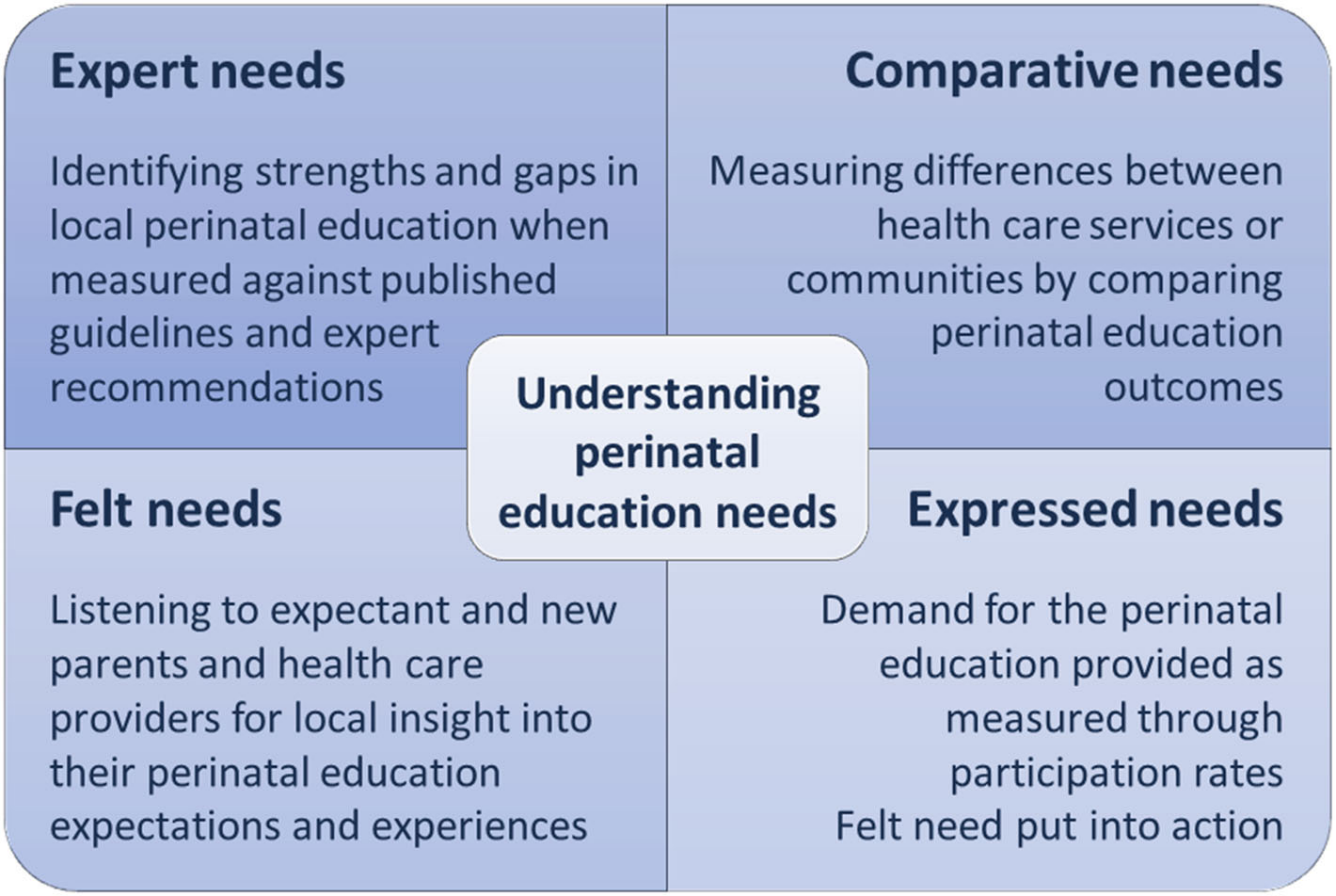
Bradshaw’s model for needs assessment applied to perinatal education

Needs assessment within perinatal healthcare should have the expectant and new parent at the centre. A needs assessment reports unmet needs (gaps in services / unmet outcomes) and assesses strengths that can be built upon and/or translated into other areas [55, 58]. To achieve the multidimensional understanding required as the first stage of this implementation project required a holistic, stakeholder centred needs assessment that considered individual and community needs within the wider context of service delivery [59]. Bradshaw’s model of need allows for this type of assessment and has proven to be an enduring and effective model used in an Australian context including perinatal care [54, 60, 61]. Figure 3 outlines how Bradshaw’s multi-layered description of need [62], can be applied to perinatal education needs assessment. How this fits within our research context is illustrated in Fig. 5.

Understanding these needs with the lens of the CFIR constructs gives focus to *what* and *how* any new innovations or changes to current perinatal education should be implemented.

#### Intervention design

The process of designing and building an evidence-based perinatal education intervention is based on the findings from the previous stage of *understanding need*. Current methods used to educate expectant and new parents are not well aligned with methods this population finds effective to seek knowledge and build skills that lead to improved health and wellbeing [13, 63]. Expectant and new parents want perinatal education that is consistent, continuous, timely and offers choice of content; is practical and covers a broad range of health and wellness topics including physical and mental health, emotional, relationship, breastfeeding and parenting skills; and, uses multiple mediums including interactive learning, peer teaching and digital media [64].

Designing a perinatal education intervention adapted to meet local needs requires two complementary strategies working in partnership (through co-design) with expectant and new parents: firstly, to understand needs within the local context and secondly, to apply of health behaviour theories in a dynamic interplay that facilitates improved health outcomes [65].

#### Applying theoretical models for behaviour change

**Fig. 4:**
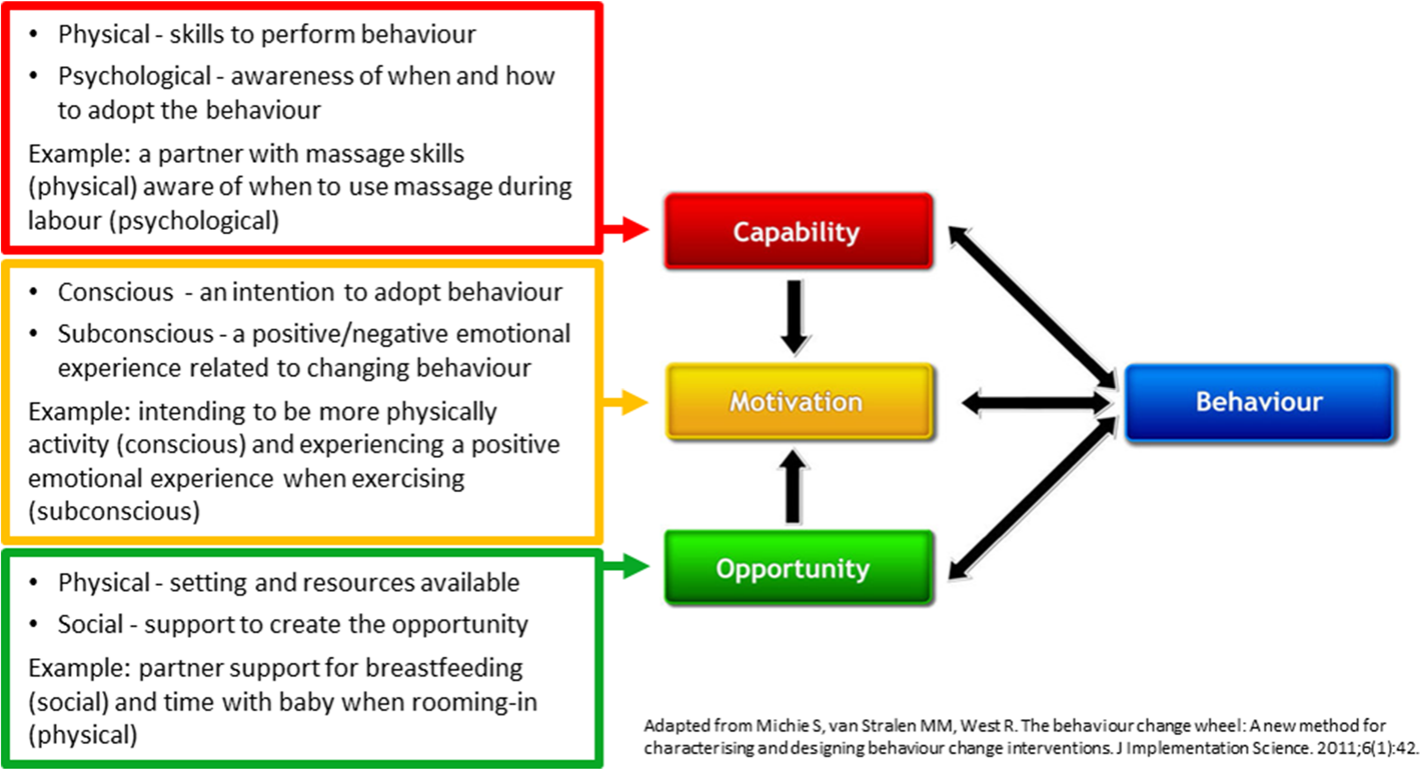
COM-B model for understanding behaviour of expectant and new parents

Theories and models for behaviour change can guide and articulate understanding of personal factors, environmental factors and behaviour and how to influence these behaviours through education interventions [66]. We have chosen the Capability-Opportunity-Motivation Behaviour (COM-B) model as a useful tool in understanding the contributors to inter-personal health behaviour [67]. The COM-B has guided the development of health interventions over recent years with many of these interventions being for expectant and new parents [32, 33]. Figure 4 gives an overview of the COM-B model and some examples of how behaviours of expectant and new parents can be understood using this model.

To build motivation, capability, and opportunity to facilitate adoption of newly gained skills, knowledge, and increased health literacy, expectant and new parents will also require self-efficacy. Self-efficacy is the belief that you can achieve an outcome or reach a desired goal [68]. Strategies that build self-efficacy considered alongside the COM-B model provide a basis for adapting and developing effective health education interventions.

Widely accepted strategies for building self-efficacy draw from the Social Cognitive Theory (SCT) described originally by Bandura [69] and have been used to inform the development of health and wellness education interventions over the last 40 years [70]. SCT suggest four methods through which self-efficacy can be developed: [71].

*Performance attainment (mastery experience):* this includes past experiences and ongoing practise to achieve mastery and successful performance. Learning through ‘doing’ is a highly effective learning method that has been successfully applied in perinatal education settings incorporating health behaviour goal setting and monitoring achievements [72, 73]. For our intervention this means practising developing skills such as pelvic floor exercises, breastfeeding, and mindfulness across multiple timepoints.

*Vicarious experience (modelling):* learning through observing others in similar experiences or performances. Hearing and seeing other parents’ lived experiences in the language of perinatal women and their partners provides a powerful form of teaching. Vicarious experience provides an opportunity for behaviour change where it may not be readily available (preparing expectant parents for labour and birth, breastfeeding, infant care and the challenges of the transition to parenthood) and improves capability without active skill practise [74]. It is also an effective motivation tool in perinatal education in anticipation of future experiences [75].

*Verbal persuasion:* this is persuasive, positive communication through education, and encouragement. It depends on the health care provider’s perceived reliability and expertise (credibility) coupled with their persuasive communication skills. Effective communication facilitates motivation and creates an opportunity for learning. Verbal persuasion can come from a variety of ‘trusted’ sources including partners, friends, health care professionals or even through tailored text messages [76].

*Physiological feedback:* this includes both physical and emotional feedback provided by physiological arousal of the autonomic nervous system and by interpretation of physical signs and symptoms (e.g., pain, fatigue, breathlessness, physical tension, and anxiety). Educators facilitate this by improving *capability* by teaching skills for identifying and self-monitoring physiological changes and then practising skills and behaviours that provide this physiological feedback. Breath awareness and mindfulness are examples that has been successfully applied in perinatal education [6]. Physiological feedback also extends to physical activity that comes from practising physical skills like movement for pain relief, exercises or partner massage and emotional feedback can also be provided through being with other parents in the same situation [63]. Physiological feedback builds subconscious motivation for adopting behaviours.

Successful perinatal education is not limited to the content or the format/s of education but also the health behaviour and communication strategies used to reach the target population. Intentionally incorporating these factors into perinatal education will contribute to success achievement of desired health and wellness outcomes. To achieving these health and wellness outcomes requires a successful implementation process.

#### Plan and execute implementation

Planning and executing the implementation of a perinatal education intervention builds on the findings from the pre-implementation stage of *understanding need* and can occur alongside the intervention design phase. Enablers and barriers to effective perinatal education delivery identified through a needs assessment define what is possible, realistic, and preferable in the local context. Co-design with health care providers is vital for engagement and contributes to the success and sustainability of the implementation.

Review of implementation research has identified key CFIR constructs (modifiable variables) that are consistently associated with implementation success [38]. These are: i) the *relative advantage* of the intervention; ii) *tension for change* within the inner setting; and iii) patients’ *needs and resources.* In addition, the local implementation *planning* process, *available resources* and the *relative priority* within the inner setting are markers of likely implementation fidelity. Understanding the local context and applying that understanding to the intentional planning is important for implementation success and a key component of implementation science models and theories like Implementation Mapping (a stepwise model for implementing health promotion interventions) [77], and PRECEDE-PROCEED (Predisposing, Reinforcing and Enabling Constructs in Educational Diagnosis and Evaluation-Policy, Regulatory and Organizational Constructs in Educational and Environmental Development) that provides an implementation road map focused on achieving health outcomes [78].

#### Models and tools to guide implementation

We have chosen models and tools that articulate with the CFIR constructs and can be operationalised in our intervention context. The CFIR-ERIC implementation strategy is designed to match construct variables to implementation strategies [50]. This tool matches the Expert Recommendations for Implementing Change (ERIC) [79] (73 implementation strategies based on expert review) directly with CFIR constructs to provide a suite of practical evidence-based implementation strategies that can be used to facilitate the implementation process.

A key component of implementation success are the individuals involved (both health care providers and patients) and their actions [80]. The Behaviour Change Wheel (BCW) provides another evidence-based guide to implementation aimed at operationalising strategies to overcoming barriers specifically related to behaviour change in individuals. The BCW uses the Theoretical Domains Framework (TDF) with the ability to characterise individual, team and organisational enablers and barriers to change mapped to the COM-B model (the central hub of the BCW) to match appropriate intervention features and policy categories to reinforce the strategies aligned to the relevant TDF domains [67].

#### Evaluate and sustain

While the CFIR and companion tools can be used for implementation evaluation, some *evaluation frameworks* solely focus on assessing process and outcomes. These frameworks provide a structure and external validity to evaluation and investigate the *why* of implementation success or failure as opposed to the determinant framework like CFIR which is primarily defining *what* variables can facilitate success (or failure) [45]. Two widely accepted standard for reporting of the evaluation of health and community interventions are PRECEDE-PROCEED and the Reach, Efficacy, Adoption, Implementation and Maintenance (RE-AIM) framework [81].

RE-AIM has been widely used in health service research [45, 82], provides extensive resources online [83], is a standardised method for reporting implementation success that is understood by researchers, funders, and policy makers [82] and fits best with the multidimensional theoretical approach we are taking. These factors make RE-AIM the preferred choice of evaluation framework for this theoretical approach. RE-AIM considers five domains of evaluation:

*Reach* is a measure of the participation by the target population and in this approach is health service data on expectant and new parent engagement with education via attendance rates and digital media usage.

*Efficacy* is the effectiveness of the interventions, this measure of impact could include proximal measures like feedback on perinatal education experience (including meeting learning needs), targeted maternal and infant health outcomes (including participant reported outcome and experience measures, and routinely reported hospital data) and quality of life measures including measures that can be translated to report economic impact. Working with expectant and new parents and other stakeholders to define what outcomes are meaningful is an important element of co-design.

*Adoption* is the uptake by health care professionals involved and provides a measure of the acceptability of the implementation process and how changes to the perinatal intervention have been actioned. Adoption can be considered at an individual level, between work units or organisation wide and provides an understanding of factors that might contribute to ongoing sustainability.

*Implementation* measures fidelity of implementation components both at the inner setting (*was it implemented into routine perinatal health care as planned?*) and at the individual patient level (*did expectant and new parents participate in the way intended?*).

*Maintenance* is a measure of sustainability either at an individual patient level (*what is the ongoing effect of the intervention?*) or within the implementation setting (*How sustainable is the practice change? What mechanisms are needed to optimise sustainability?*). This measure will feed into developing a mechanism for an ongoing cycle of improvements based on responding as feedback is received and needs change (e.g., when new guidelines emerge, or local outcomes are not as expected).

## Results

**Fig. 5:**
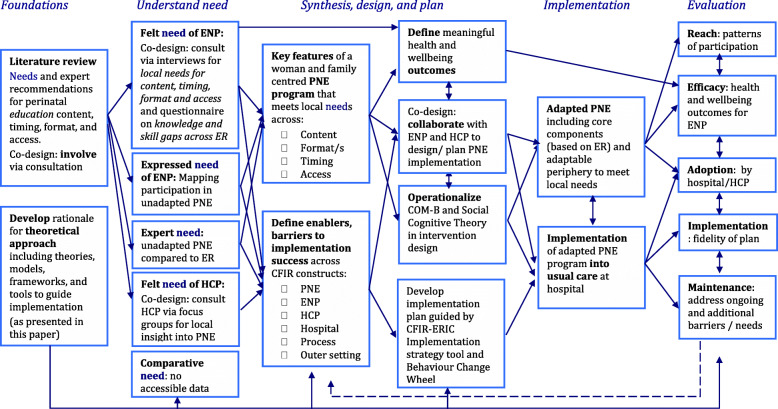
Applying a multidimensional theoretical approach to perinatal education design and implementation within a maternity hospital. CFIR constructs: PNE = perinatal education program (intervention - unadapted and adapted), ENP = expectant and new parents (patient), HCP = Health care providers (individuals involved); ER = expert recommendations (outer setting); Hospital (inner setting) and Process

The six elements above come together to form a theory-informed approach to the implementation of a perinatal education intervention within a large maternity hospital (Fig. 1). The practical application and interactions between each of these elements throughout our research is shown in Fig. 5 in a schematic diagram illustrating the content, processes, and linkages of steps to design and implement a perinatal education program intervention within a large maternity hospital.

## Discussion

In addition to CFIR, there are complementary theories, models, frameworks and tools that fit well in this approach when they are viewed through a CFIR lens that is modified for this context. In the development and planning phase of an implementation project it is important to understand local needs. Bradshaw’s model provides a holistic approach for considering need across the CFIR domains. Elements of the BCW/COM-B and SCT contribute to the evidence-based *intervention design* by providing methodology for delivering education that will influence health behaviours. Through the *implementation* process, the CFIR -ERIC matching tool and the BCW provide useful methodologies for planning and executing implementation. Finally, in *evaluation*, the RE-AIM domains provide a methodology for assessing and reporting implementation outcomes including developing a plan for *sustainability*. This multidimentional theoretical approach is applied in partnership with the key stakeholders throughout by *co-designing* with expectant and new parents and health care providers.

One criticism to date in the operationalisation of implementation science is that the rationale for using a combination of theories, models, frameworks, and tools in a study is often unclear and may inadvertently create unnecessary complexity or redundancy [25, 46]. Curating and forming a cohesive multidimensional methodology to this health service research not only addresses our own study purposes but provides a rationale for a theory-informed approach that can be operationalised and tested across a range of other contexts.

This paper presents a theoretically underpinned approach to designing and implementing a woman and family centred perinatal education program guided by CFIR. Through understanding, utilising and reporting the rationalle behind this implementation science approach and subsequent evaluation we aim to meaningfully improve the perinatal care provided in our local setting and meet key requirements of implementation science research including reporting guidelines [28, 84].

## Conclusion

Developing, delivering, and evaluating an education intervention for expectant and new parents within a large maternity hospital first required the understanding and synthesis of the implementation science theories, models, frameworks, and tools that can be operationalised to facilitate implementation success and intervention sustainability. In this worked example this has facilitated a deep and collaborative understanding of the needs of expectant and new parents, the local enablers and barriers to successful implementation and the tools to meet these needs, leverage enablers and address barriers. Lessons learnt through comprehensive evaluation will facilitate improvement and sustainability of this intervention and future local health service change.

## Data Availability

Not applicable.

## Abbreviations

CFIR: Consolidated framework for implementation research
iPARIHS: Integrated promoting action on research implementation in Health Services
NASSS: Non-adoption, abandonment and barriers to scale-up, spread and sustainability
IAP2: International association for public participation
COM-B: Capability, opportunity, motivation behaviour model
SCT: Social cognitive theory
PRECEDE-PROCEED: Predisposing, reinforcing and enabling constructs in educational diagnosis and evaluation-policy, regulatory and organizational constructs in educational and environmental development
ERIC: Expert recommendations for implementing change
BCW: Behaviour change wheel
TDF: Theoretical domains framework
RE-AIM: Reach, effectiveness, adoption, implementation and maintenance
